# A new deep-sea balanomorph barnacle (Cirripedia: Thoracica: Bathylasmatidae) from Chile

**DOI:** 10.1371/journal.pone.0197821

**Published:** 2018-06-13

**Authors:** Juan Francisco Araya, William Anderson Newman

**Affiliations:** 1 Programa de Doctorado en Sistemática y Biodiversidad, Departamento de Zoología, Facultad de Ciencias Zoológicas y Oceanográficas, Universidad de Concepción, Concepción, Chile; 2 Centro de Investigaciones Costeras de la Universidad de Atacama (CIC-UDA), Universidad de Atacama, Copiapó, Región de Atacama, Chile; 3 Scripps Institution of Oceanography, University of California-San Diego, La Jolla, CA, United States of America; Laboratoire de Biologie du Développement de Villefranche-sur-Mer, FRANCE

## Abstract

Deep waters of the South Pacific off northern Chile remain poorly studied, particularly in regard to invertebrate faunas. Some recent works include new records on deep-water species, mostly from the bycatch of benthic fisheries concentrated along the continental margin of the country. Among these, a few specimens of an unidentified bathylasmatine balanomorph were collected off Caldera, northern Chile, and they are described here as *Bathylasma chilense* sp. nov. While this is the second report of a bathylasmatid in the Eastern Pacific Ocean, the first being *Tetrachaelasma southwardi* Newman & Ross, 1971, it is not only the first but the deepest known (1800–2000 m) species of *Bathylasma*. Its discovery increases the number of described *Bathylasma* species to eight, four of which are extant. This is the third deep-water balanomorph cirriped recorded for the region where it may represent an isolate from a West Wind Drift fauna, an immigrant from the western Pacific, or a relict of a once cosmopolitan Paleocene-Eocene fauna now having an amphitropical component.

## Introduction

Cirripeds are basically attached, setose-feeding crustaceans frequently encountered in high densities in the intertidal and coastal inshore shallow waters [1]. Most have six free-living planktotrophic naupliar stages and a non-feeding cyprid stage that selects the place to settle and metamorphose into the juvenile. Balanomorph cirripeds, the most species rich group of thoracic barnacles, are members of the suborder Balanomorpha Pilsbry, 1916 (sensu Koči et al. [2]). This group encompasses sessile barnacle species having a symmetrical wall formed of eight, six, or four plates or a whole solid or concrescent shell, and an operculum formed by paired terga and scuta, except in *Xenobalanus* Steenstrup, 1852, where the operculum has been lost [3, 4]. The balanomorphs include a large diversity of species largely found in shallow waters around the world; they are usually gregarious, often found in large communities along the shore where they may represent important components of many coastal ecosystems [5].

Some difficulties arose among the balanomorphs some time ago, in assigning some species of the genus *Balanus* (*B*. *corolliformis* Hoek, 1883 and *B*. *hirsutus* Hoek, 1883) to either the families Balanidae and Chthamalidae due to shared characteristics of the parietes, the cirri and the absence of a notched labrum [6]. The characteristics of two new species allowed Hoek [6] to propose the new genus *Hexelasma* for them (*H*. *velutinum* and *H*. *arafurae*) plus *B*. *callistoderma* Pilsbry 1911 and *B*. *hoekianus* Pilsbry 1911) even though both lacked a notched labrum and had a longer ramus of the third cirrus, he included them in his new genus in the family Balanidae. Pilsbry [7] reviewed this group of deep-water barnacles, comparing *Hexelasma* to the chthamalid genus *Pachylasma* (on the basis of the comparable arrangement of the wall plates and nonbalanid nature of the appendages), and concluded that the form of the labrum and of the third cirrus established *Hexelasma* as a member of the Chthamalidae rather than Balanidae. He also described the new species *Hexelasma americanum* and returned *H*. *hoekianus* to *Balanus*, designating it as the type species of the newly proposed subgenus *Metabalanus* Pilsbry [7]. Some doubts were expressed about this classification by Bage [8] after examining the species *H*. *antarticum*, which she thought belonged to Balanidae rather than Chthamalidae, apparent from similarities of the soft parts while Utinomi [9] concluded the contrary.

The discovery of longitudinal tubes secondarily filled with a translucent, yellow, chitinous material permeating the parietes of *H*. *callistoderma* and *H*. *americanum* led Newman and Ross [3] to re-consider the existing definitions for the families Chthamalidae and Balanidae, concluding that the so-called “hexelasmoids” required a new familial-level taxon to accommodate them in the Balanomorpha. The new family, the Bathylasmatidae, including the genera *Aaptolasma* nov., *Hexelasma* Hoek, *Tessarelasma* Withers, *Tetrachaelasma* nov. as well as *Bathylasma* nov., was coordinate with the families Balanidae, Chthamalidae and Tetraclitidae. It was diagnosed as having the crest of labrum without notch; one or both rami of second and third cirri sometimes antenniform; and a wall solid or permeated by a single row of chitin-filled longitudinal canals and without radii. Furthermore, they placed the Bathylasmatidae in the superfamily Balanomorphoidea (replacement name, Coronuloidea according to Newman & Ross [10]), and divided it into two subfamilies, the Bathylasmatinae, containing the genera *Bathylasma*, *Tessarelasma* and *Tetrachaelasma*, and the Hexelasmatinae containing *Aaptolasma* and *Hexelasma* [11]. Foster [12], who followed Pilsbry (1916), maintained that *Pachylasma* and *Hexelasma* were closely related, and thus transferred *Bathylasma* and *Mesolasma* Foster from Bathylasmatidae to the family Pachylasmatidae upon shell construction, a family later included in the superfamily Pachylasmatoidea by Buckeridge [13]. Jones [14] maintained the separation of the genera *Bathylasma* together with genera *Mesolasma*, *Tessarelasma* and *Tetrachaelasma* in a separate subfamily than *Hexelasma*, while retaining all of them in the Pachylasmatidae. Buckeridge and Newman [15], however, following Newman [16], ranked the Bathylasmatidae under the superfamily Tetraclitoidea, a proposition supported by Tsang et al. [17] in its molecular phylogeny of the acorn barnacle family Tetraclitidae, and more recently by Chan et al. [4] in their molecular phylogeny of the lower acorn barnacle families.

The genus *Bathylasma* Newman & Ross, 1971, the type genus of the family Bathylasmatidae, is currently characterized by having a membranous basis, a wall of six generally thin parietal plates with well-developed alae but lacking radii, terga and scuta with prominent articular ridges, and a quadridentoid mandible and ctenopod cirri (setae arranged in rows along each ramus). Prior to the discovery of the new species off of northern Chile, the genus encompassed three extant species, one in the south-western Pacific, one circum-Antarctic and one in the north-eastern Atlantic Ocean. Four fossil species are also known, two in the vicinity of New Zealand, one in Australia and one in North Carolina, USA; the oldest records for the genus being Upper Eocene and Paleocene in the northern and southern hemispheres respectively [18, 19]. In the present work, as part of ongoing studies reviewing macro-invertebrates from the bycatch fauna of commercial fisheries off northern Chile, which have revealed several new species and new records for the south-eastern Pacific [20–26], we present the first record of the genus *Bathylasma* Newman & Ross, 1971 in the Eastern Pacific Ocean. It also constitutes the deepest unequivocal record for the genus.

## Material and methods

### Sample and morphological examination

**Fig 1:**
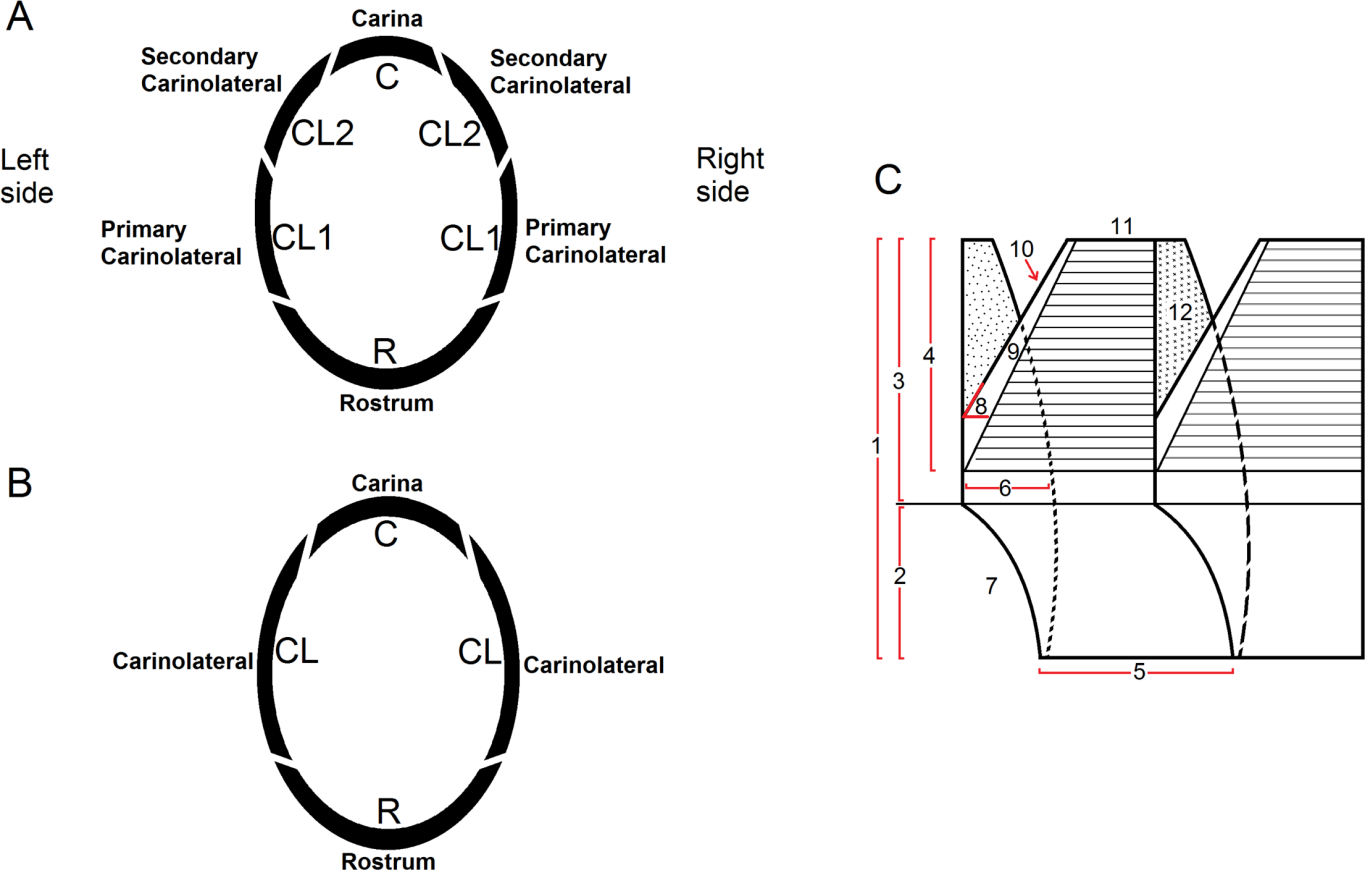
Scheme of wall construction and plate nomenclature in Bathylasmatidae genera. A, plan view for *Bathylasma* Newman & Ross, 1971, *Hexelasma* Hoek, 1913 and *Mesolasma* Foster, 1981. B, plan view for *Tetrachaelasma* Newman & Ross, 1971. C, plate nomenclature, showing schematic left lateral carinolateral wall plate between adjacent plates, and viewed from within. 1: total height; 2: height below sheath; 3: height of entire sheath; 4: height of sheath crossed by growth lines; 5: width of basal margin; 6: width of ala; 7: inferior alar margin; 8: alar angle; 9: secondary alar increment or welting; 10: superior alar margin; 11: apex; 12: portion of wall overlapping adjacent plate (in this case without radii, a radius being the exterior counterpart of the welting (9) and serving much the same function) (modified from Newman & Ross [3]).

The specimens of the new species were collected attached to an unidentified zoantharian entangled in a longline from between 1800–2000 m depth, as bycatch from the fishery for the Chilean Seabass, *Dissostichus eleginoides*, from about 32 km off Caldera (26°44’ S; 71°07’ W), Región de Atacama, northern Chile. As this material was serendipitously collected in the fish bycatch, no permit was necessary for the current research. Cirripeds are not endangered nor protected by local law. The specimens were examined by light and scanning electron microscopy (SEM), and measured with vernier callipers reading to two decimal places. The appendages were mounted on glass slides in glycerine jelly for illustration. For the morphometric analysis, the works of Newman & Ross [3] and Jones [14] were followed. The systematic arrangement follows Newman & Ross [3] and Newman [16], as modified by Koči et al. [2]; the interesting but presently intractable complications introduced by the genetics of Tsang et al. [17] for the present being ignored. A scheme for the wall construction (plan views) and for plate nomenclature in Bathylasmatidae genera is presented in Fig 1.

### Deposition of types

The holotype is deposited in the collections of the Museo Nacional de Historia Natural, at Santiago, Chile (MNHNCL CIR-15057), the first paratype is deposited in the Benthic Invertebrate Collection of the Scripps Institution of Oceanography, San Diego, USA (SIO/BIC Cat. No.12199) and the second paratype in the collection of the Museo Paleontológico de Caldera, in Caldera, Chile (MPCCL020518).

### Nomenclatural acts

The electronic edition of this article conforms to the requirements of the amended International Code of Zoological Nomenclature, and hence the new names contained herein are available under that Code from the electronic edition of this article. This published work and the nomenclatural acts it contains have been registered in ZooBank, the online registration system for the ICZN. The ZooBank LSIDs (Life Science Identifiers) can be resolved and the associated information viewed through any standard web browser by appending the LSID to the prefix “http://zoobank.org/”. The LSID for this publication is: urn:lsid:zoobank.org:pub: F7B5CB72-7DFA-48E2-98B1-0388472A5DEB. The electronic edition of this work was published in a journal with an ISSN, and has been archived and is available from the following digital repositories: PubMed Central, LOCKSS.

## Results

### Systematics

Subclass Cirripedia Burmeister, 1834

Superorder Thoracica Darwin, 1854

Order Balanomorpha Pilsbry, 1916 = Sessilia Lamarck, 1818 [2]

Infraorder Balanoformes Koči, Newman & Buckeridge, 2017

Family Bathylasmatidae Newman & Ross, 1971

### Key for identification of extant genera of the Bathylasmatidae

1. Six wall plates. . . .. . . .. . . .…………………………………………………………………….2

- Four wall plates………………………………. . . ..*T**etrachaelasma* Newman & Ross, 1971

2. Parietes solid, without chitinous laminae and/or strips………………………………….3

- Parietes with chitinous laminae and/or strips; basis calcareous, thin, rarely centrally membranous *Hexelasma* Hoek, 1913

3. Basis calcareous *Mesolasma* Foster, 1981

- Basis membranous *Bathylasma* Newman & Ross, 1971

Genus ***Bathylasma*** Newman & Ross, 1971

**Diagnosis:** Adult wall of six relatively thin, calcareous compartmental plates (R-CL1-CL2-C); rostrum compound (not tripartite). Parietes with prominent horizontal growth ridges lined with small setae; chitinous laminae absent. External alar ridges diverging from inferior alar margin; superior alar margin with welting. Basis membranous. Articular ridges of tergum and scutum prominent. Mandible quadridentoid. Caudal appendages absent (modified after Jones [14]).

**Type species:** *Balanus corolliformis* Hoek, 1883, by original designation.

**Fig 2:**
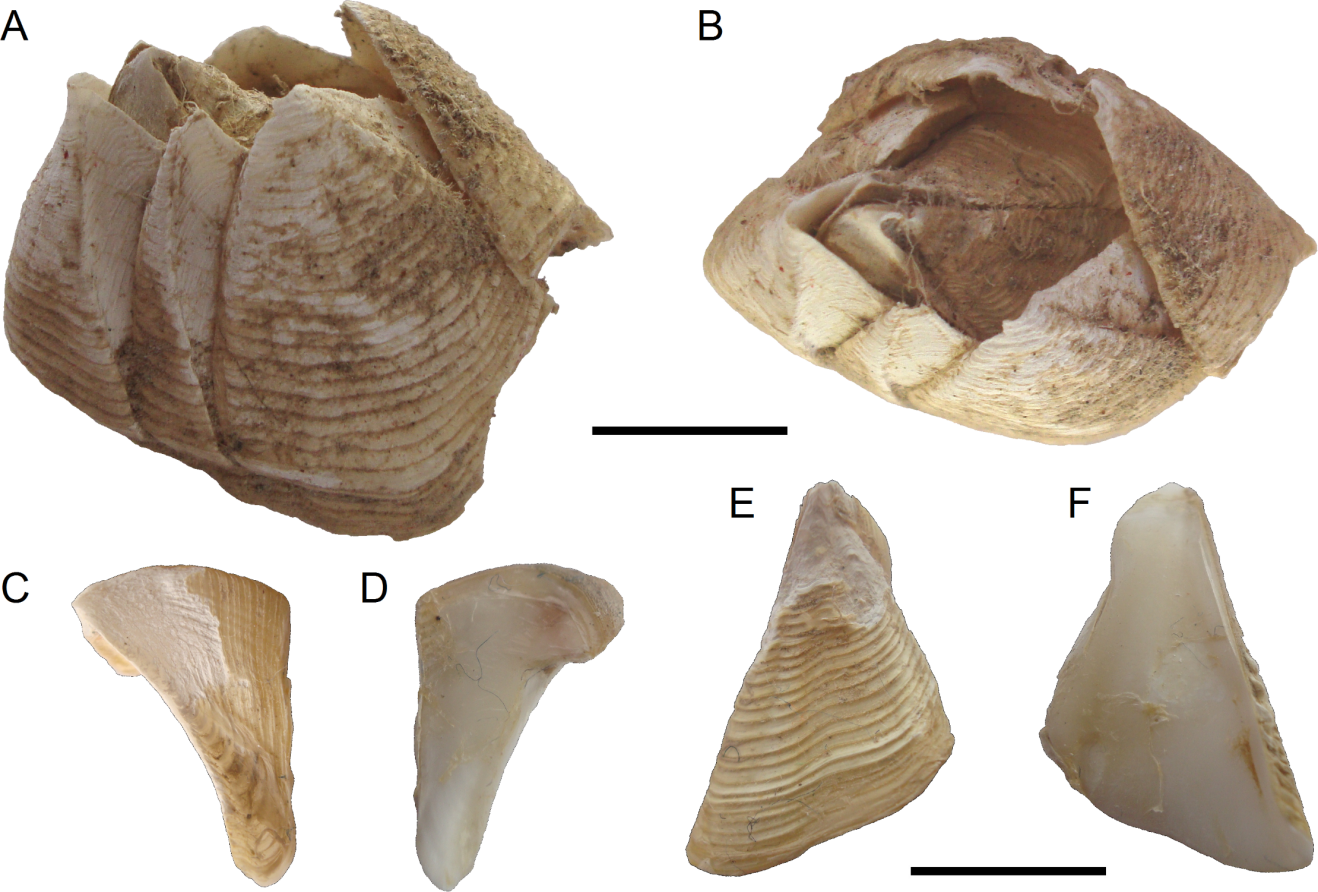
*Bathylasma chilense* sp. nov., holotype. A, complete specimen side (left) view. B, apical view. C, external view of tergum. D, internal view of tergum. E, external view of scutum. F, internal view of scutum. Scale bar is 10 mm for A and B, and 5 mm for C–F.

**Fig 3:**
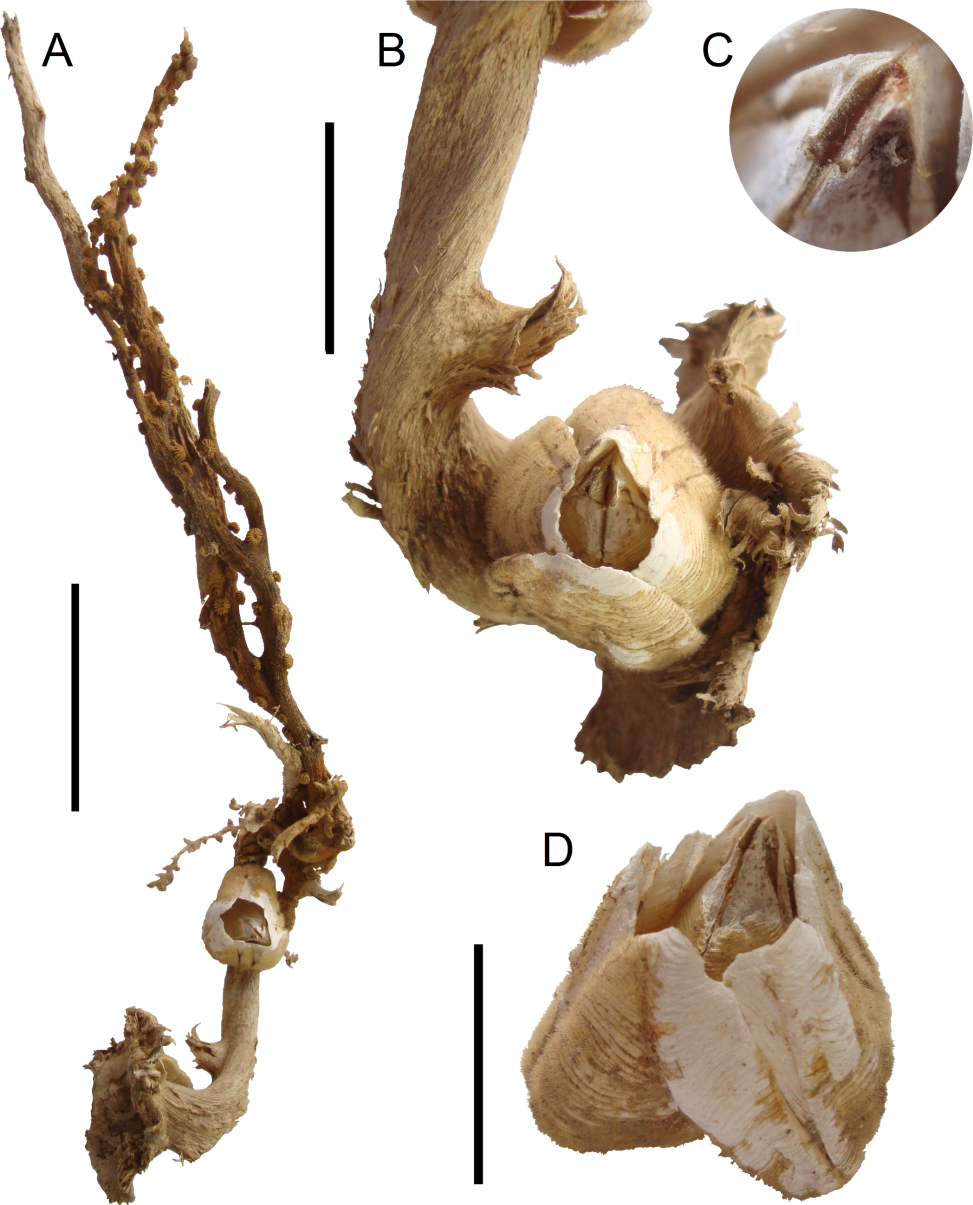
*Bathylasma chilense* sp. nov., paratypes. A, specimens in situ on unidentified zoantharian. B, detail of paratype 1. C, detail of tergal notch. D, oblique view of paratype 2. Scale bars are 50 mm for A, 20 mm for B, 5 mm for C and10 mm for D.

**Fig 4:**
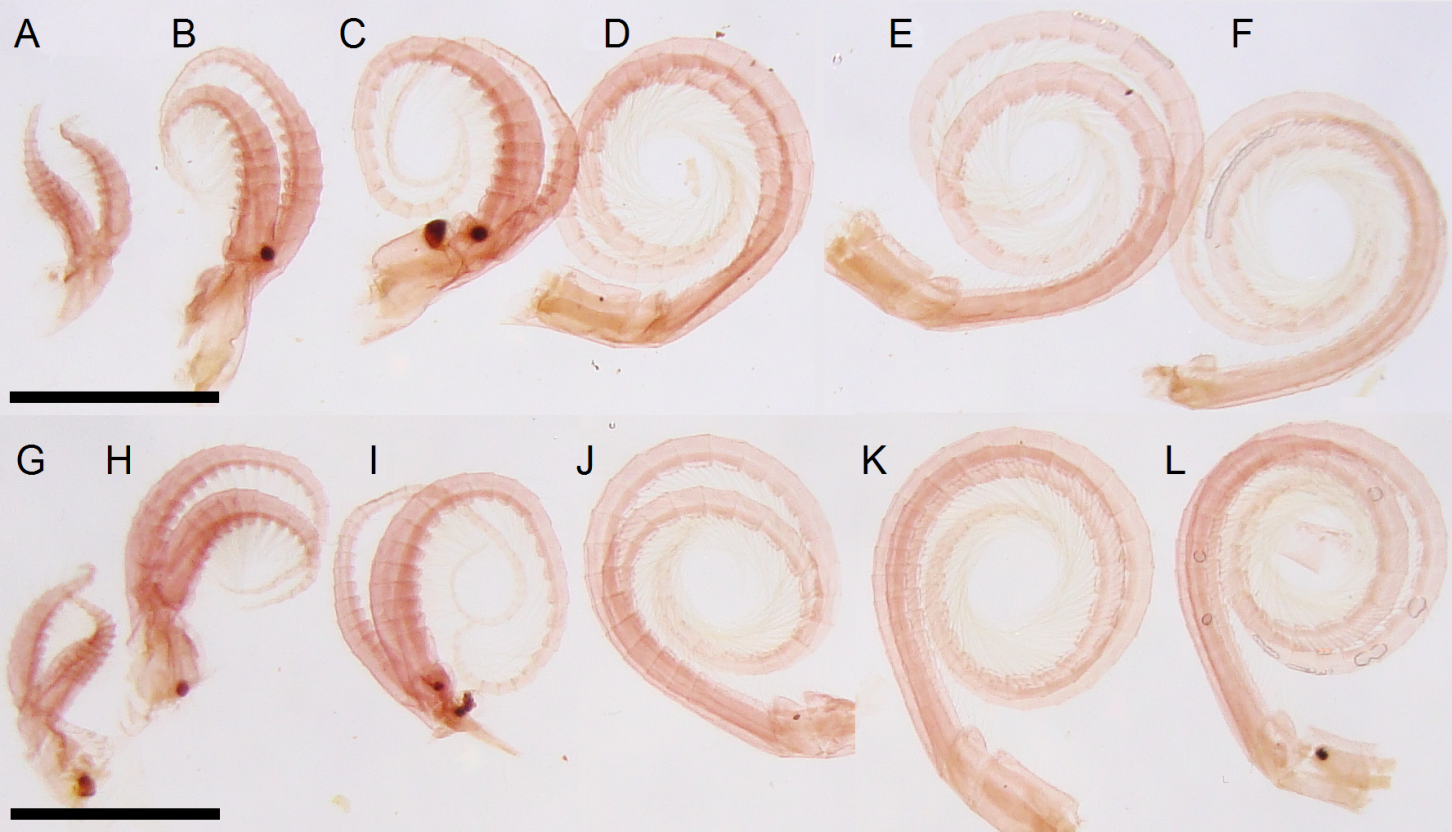
*Bathylasma chilense* sp. nov., holotype. A-F: Right cirri. A, cirrus I. B, cirrus II. C, cirrus III. D, cirrus IV. E, cirrus V. E, cirrus VI. G-L: Left cirri.G, cirrus I. H, cirrus II. I, cirrus III. J, cirrus IV. K, cirrus V. L, cirrus VI. Scale bars are 5 mm.

**Fig 5:**
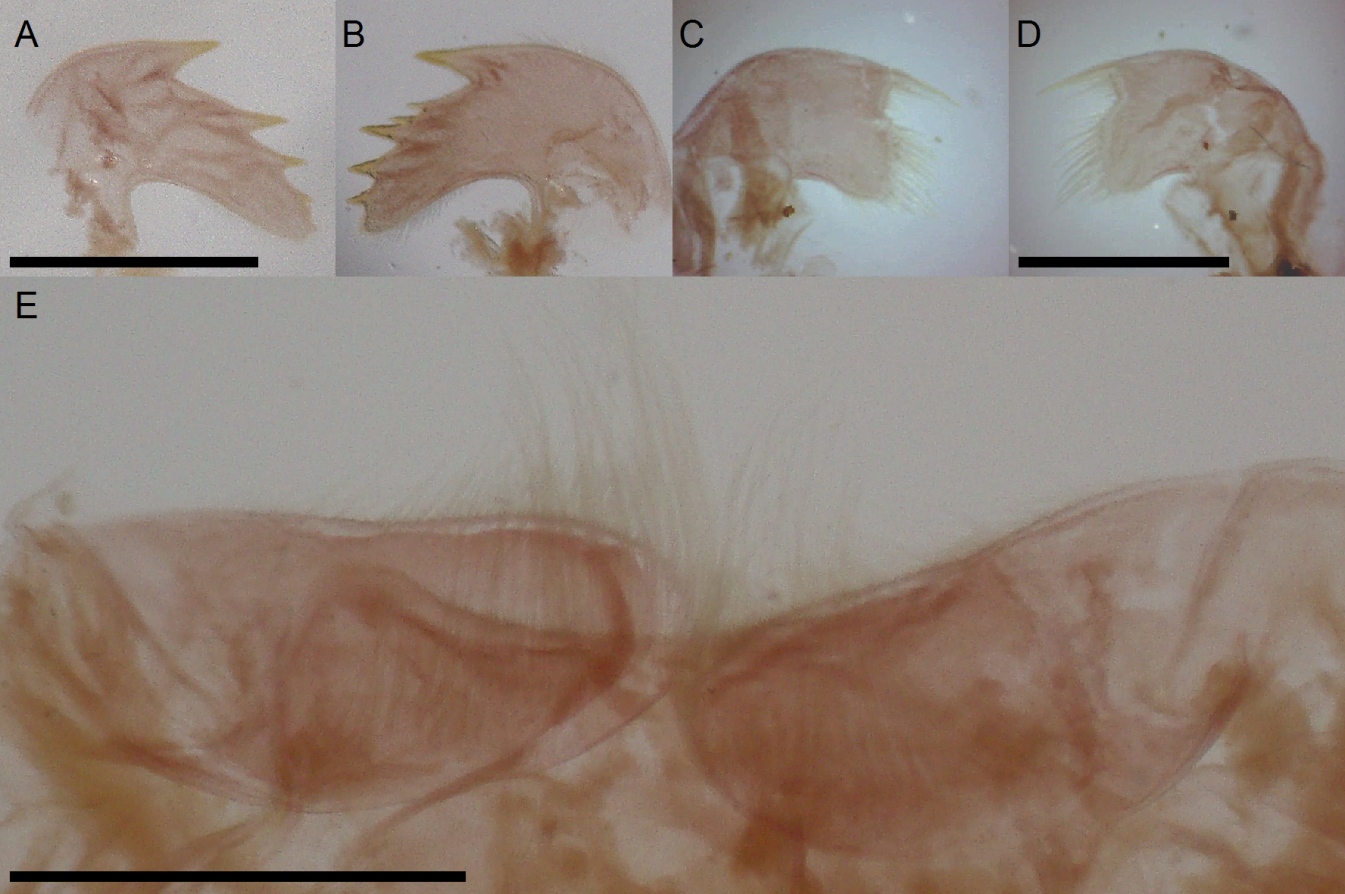
*Bathylasma chilense* sp. nov., holotype. A, right mandible. B, left mandible. C, right maxillule. D, left maxillule. E, labrum and mandibular palps. Scale bars are 1 mm.

**Fig 6:**
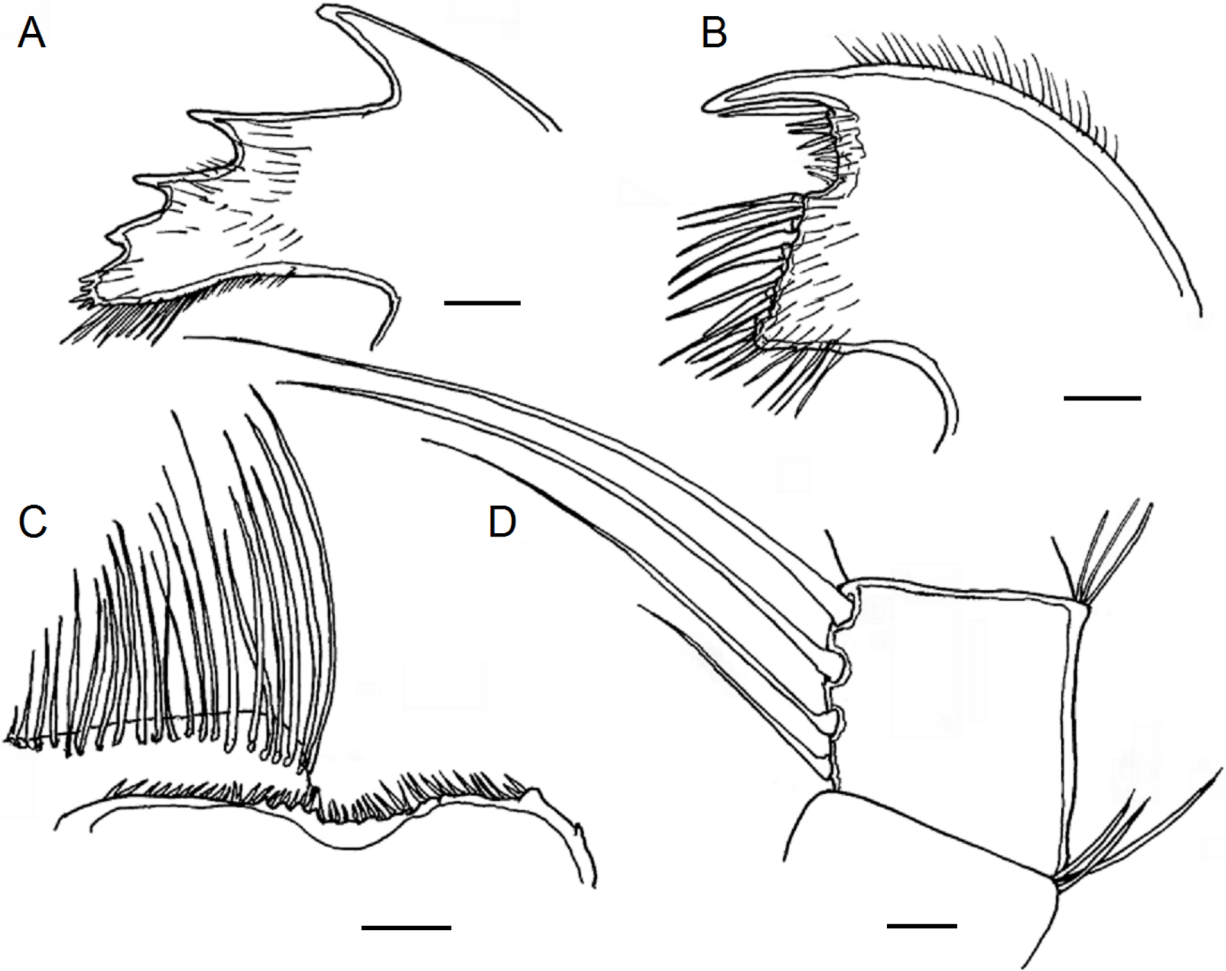
*Bathylasma chilense* sp. nov., paratype 1. A, crest of labrum and right palp (left palp deleted). B, right mandible. C, right first maxilla. D, intermediate segments of cirrus VI (one seta of each of the normally paired setae on the lesser curvature of the complete segment deleted). Scale bars are 0.125 mm.

***Bathylasma chilense* sp. nov.** (Figs 2–6)

urn:lsid:zoobank.org:act:E6B7B9C4-107D-4CE7-B1A2-BEE8F85509F3

**Diagnosis:** Parietal plates not flaring or slightly flaring at the orifice; prominent rows of setae on prominent external growth ridges. Basal width of CL2 about 25% basal width of CL1. Conspicuous welting along superior alar margins; growth lines not parallel with inferior alar margin, leaving smooth, triangular extension of internal alar surface beneath growth lines on outside, towards alar angle. Scutum with externally regular, well-spaced growth lines decorated with setae, not cut by longitudinal folds, basal margin about 75% length of occludent margin, articular ridge projecting. Tergum with articular ridge projecting slightly beyond articular margin, spur narrowly rounded, about 25% width of basal margin, with 7–8 strong muscle crests not projecting beyond the basal-tergal margin. Typical cirri and mouth parts distinctly reddish-brown to amber in color.

**Material examined:** Holotype: MPCCLXXXXA, dissected, disarticulated (Fig 2A–2F); paratype 1 (SIO/BIC Cat. No 12199), partly disarticulated (Figs 3B, 6A–5D); paratype 2 (MPCCLXXXXB), articulated specimen (Fig 3A and 3D). All material is from the type locality.

**Type locality:** Deep waters (1800–2000 m) off Caldera (26°44’ S; 71°07’ W), Región de Atacama, northern Chile.

**Description:** The holotype of *Bathylasma chilense* sp. nov. is moderately large in size, with a rostro-carinal diameter of 31.2 mm and a carinal height of 13.9 mm (Fig 2A and 2B). The conical wall of parietal plates gently slopes at an angle between 45°-60° from the base. The solid parietal plates are chalky white, with slightly irregular growth ridges covered by a thin epidermis with numerous hair-like pale brown chitinous bristles in bundles along the growth lines. The inner surface of the parietes is matte white, porcellaneous and smooth in their lower halves, and the sheath in their upper part is crossed by fine horizontal lines. The rostral plate is broad and convex, with a basal width about equal to that of the carinal plate. The carina is slightly flared; the secondary carinolateral plates are narrow, of 33–50% of the width of the basal margin of primary carinolateral plates. As seen from the exterior, the alae are sharply delimited from the parietal portion of the plate and usually exposed about 66% of the height of the wall, below which point the parietal portions of the plates come into contact. External alar growth lines parallel the inferior alar margin and then diverge abruptly near alar angle. Sheath noted above slightly overhanging the interior of the compartment; crossed horizontally by fine striae occupying 50% the length of plate. Basis membranous, very thin.

Orifice small, ovate-rhomboidal in outline. Opercular plates lodged upright in orifice. Scutum (Fig 2E and 2F) larger and broader than tergum; triangular, elongated, slightly concave in the middle; length of convex basal margin about 75% length of occludent margin; articular margin curved toward occludent margin; externally growth ridges regular, slightly sinuous and covered with minute but noticeable setae, without radial striations; apex blunt, incurved; internally adductor muscle pit large, shallow, marked whitish; situated about 50% the length of valve; lateral depressor muscle pit barely visible, whitish. Tergum (Fig 2C and 2D) more or less triangular, much wider than scutum, curved and thickened towards articular margin; covered with a yellowish cuticle sculptured by fine growth lines and a few axial lines, articular margin with growth ridges raised to form elevated ridge; internally with 7–8 distinct, marked and prominent muscle attachment crests for depressor muscle crest not projecting beyond basal margin; inner surface smooth. Articular furrow marked, narrow; tergal spur rounded, about 25% of the width of basal margin. Tergal notch large and deep (Fig 3C). The interior margin of the apical parts of scutum and tergum lined with fine dark-brown setae.

Labrum with shallow median depression marked by low, rounded teeth. Mandibular palps oblong, with fine setae along their inner edge (Fig 5E). Mandible quadridentoid, first tooth the largest, separated from second and third, inferior angle dentate, inferior angle with five or so short spines; left mandible with two strong spines between first and second tooth (Fig 5A and 5B). Maxillule setose; two long, stout setae above and followed by 3–4 pairs of smaller setae; straight, stepped cutting margin below with four or five pairs of larger setae; inferior angle small, with three pairs of smaller setae (Fig 5C and 5D). Cirrus I with unequal rami; anterior ramus slightly longer than posterior; both rami with segments setose. Articles wider than high; the anterior surface of each segment is produced in both rami and the projections bear a tuft of spines at their summits. Cirrus II longer than cirrus I; rami unequal, anterior ramus slightly shorter than posterior ramus; all segment setose, articles wider than high and slightly protuberant along the anterior margins. Cirrus III longer than cirrus II; rami unequal; anterior ramus slightly shorter and thinner than posterior ramus, which is much wider; all segment with setae, neither ramus is antenniform. Segments becoming oblong distally, wider than high; slightly protuberant along the anterior margins. Posterior ramus with 3–4 pairs of long setae on anterior faces of distal segments, posterior segments densely setose; anterior ramus with 3–4 pairs of long setae on anterior faces of distal segments, proximal segments with 4 pairs. Cirrus IV to VI similar, longer than cirrus III; rami subequal, segments oblong, with 3–4 pairs of long setae on anterior faces (Fig 4A–4L). Cirral formula is provided in Table 1 as follows:

**Table 1:** Segment counts of *Bathylasma chilense* sp. nov.

Specimen	Cirri	I	II	III	IV	V	VI
Holotype	left/right	12/13	25/21	32/29	32/30	35/36	35/30
Paratype 1	left/right	14/15	19/24	30/29	30/29	33/36	34/31

Penis slightly shorter than length of cirrus VI; finely annulated and indistinctly hairy.

**Habitat:** The specimens were found attached to stem and branches of an unidentified arborescent parazoanthid (Fig 3A), among the incidental (bycatch) fauna entangled in a longline off Caldera (26°44’ S; 71°07’ W) at about 1800–2000 m depth, January 24, 2015, by the fishing vessel Rocio III (Caldera, Chile).

**Etymology:** Named for where discovered: Chile, the first Eastern Pacific representative of the genus.

**Remarks:** *Bathylasma chilense* sp. nov. is rather variable in its outline, the three specimens ranging from ovate to triangular when viewed from above, with differences in curvature involving the carina and rostrum. We assume this variation is related to the habitat, living in the branches of a parazoanthid. The carinolateral plates are much reduced in width in the two paratypes. The wall plates are weakly articulated as they are in *B*. *corolliforme* (Hoek, 1883) collected from near Heard and MacDonald Islands [27]. Of interest is that the soft parts are a uniform reddish-brown to amber color. While this differs from the other species of the genus as well as most other species in the family, *Tetrachaelasma tasmanicum* Buckeridge, 1999 was reported as having similar reddish-brown soft tissues [28]. No complemental males—recorded as occurring in the exposed apically articular furrow of the tergum by Foster [29] and Dayton et al. [30] for *B*. *alearum* and *B*. *corolliforme*—were observed in the present specimens.

## Discussion

*Bathylasma* Newman & Ross, 1971 currently contains three extant deepwater species having spatially isolated (allopatric) distributions: 1) *Bathylasma hirsutum* (Hoek, 1883) in the Northeast Atlantic, ranging from the Faroe Islands south to the Azores at 944–1829 m of depth [12, 31], 2) *Bathylasma corolliforme* (Hoek, 1883) from the circum-Antarctic at depths of 50–1464 m [3], and 3) *Bathylasma alearum* (Foster, 1978) from New Zealand and eastern Australia northwards to Vanuatu at depths between 414–1750 m [12]. The genus is also represented by four fossil species, *Bathylasma aucklandicum* (Hector, 1888) from the Oligo-Pliocene of New Zealand, *Bathylasma costatum* Buckeridge, 1985 from the Lower Miocene of Victoria, Australia [19], *Bathylasma corrugatum* Zullo & Baum, 1979 from Upper Eocene of North Carolina, USA [18], and *Bathylasma rangatira* Buckeridge, 1983 from the Lower Palaeocene to Lower Eocene of the Chatham Islands [13, 14, 32]. The new species, *Bathylasma chilense* from northern Chile, is compared to other extant species of the genus *Bathylasma* in Table 2, which forms the basis for the key below. Therefore only a brief comparison is given here; it differs from its recent congeners as follows: 1) from *B*. *alearum* of New Zealand in having a much smaller carinal plate and a much wider tergum with a more elongated and curved spur; 2) from the circum-Antarctic species *B*. *corolliforme* in having smaller and less flaring parietal plates and smaller carinolaterals, a smaller orifice which is smaller than its base; a less curved scutum with very narrower growth-ridges in the superior half of the valve, and a much shorter tergum, with a distinct spur. *Bathylasma hirsutum* has a larger carinal margin and a more curved carina and a more marked articular ridge in the tergum than in the new species. One or the other mandible of *B*. *chilense* sp. nov. apparently differs from all the other *Bathylasma* species in having two strong spines between the first and second teeth and in lacking small subsidiary cusps on upper or lower margins of tooth 4. The new species differs from the fossil species *B*. *aucklandicum* by the presence of a distinct sheath, in having a larger tergal spur and in lacking the tall, thin and cylindrical shell of this species, which can reach up to 18 cm in height [19]. *Bathylasma costatum* differs from the new species in having much shorter, broader parietes, without strong vertical ribbing. It also differs from *B*. *corrugatum*, the second oldest representative of the genus [18], in lacking the regularly spaced and prominent corrugations of the alae, and from the fossil *B*. *rangatira* Buckeridge, 1975, in lacking the chevron-shaped growth striae on the parietes, and its smaller size (*B*. *rangatira* having a length up to 54 mm high), much thinner shells, and flat rather than convex scuta [33].

**Table 2:** Morphological characters of extant *Bathylasma* species.

	Aperture and parietal plates	Alar growth lines	CL1:CL2	Tergum	Alar welting	Scutum shape	Scutum growth lines	Mandible
***Bathylasma alearum* (Foster, 1978)**	Not flaring; prominent setae on prominent external growth lines	Not parallel to inferior alar margin	Basal width CL2 = 25% basal width CL1	Artic. ridge projecting well beyond artic. Margin; spur about 14% width basal margin, set at 25% basal margin from b-s angle; 8–9 muscle crests proj. Just below basal margin	Conspicuous; narrow	Basal margin 70% occludent margin; artic. ridge proj. beyond artic. margin	Well-spaced, regular; not cut by longitudinal folds.	Quadridentate; teeth 2–4 with small side teeth; upper and lower margins of tooth 4 with small subsidiary cusps
***Bathylasma chilense* sp. nov.**	Not flaring, short setae on membrane above coarse external growth lines	Not parallel to inferior alar margin	Basal width CL2 = 50% to 33% basal width CL1	Artic. ridge projecting slightly beyond artic. margin; spur narrowly rounded, set at b-s angle; 7–8 muscle crests not proj. below basal margin.	Conspicuous	Basal margin about 75% of occludent margin; artic. ridge proj. beyond artic. margin	Well-spaced, irregular; not cut by longitudinal folds.	Quadridentate; without any subsidiary cusps; with two strong teeth between first and second teeth
***Bathylasma corolliforme* (Hoek, 1883)**	Flaring, short setae on membrane above coarse external growth lines	Parallel to inferior alar margin	Basal width CL2 = 10% basal width CL1	Artic. ridge projecting well beyond artic. Margin; spur indistinct, set at b-s angle; 8–9 muscle crests not proj. below basal margin.	Conspicuous; narrow	Basal margin 75% occludent margin; artic. ridge slightly proj. beyond artic. margin	In upper half well-spaced, narrow in lower half; regular; not cut by longitudinal folds	Quadridentate; teeth 2–4 with small side teeth; upper and lower margins of tooth 4 with small subsidiary cusps
***Bathylasma hirsutum* (Hoek, 1883)**	Not flaring, small setae on fine external growth lines	Not parallel to inferior alar margin	Basal width CL2 = 14% basal width CL1	Artic. ridge projecting beyond artic. margin; spur 17% width basal margin, set at 8% basal margin from b-s angle; muscle crests not proj. below basal margin.	Almost absent; very narrow	Basal margin 63% occludent margin; artic. ridge proj. beyond artic. margin	Many, well-spaced, regular; not cut by long folds	Quadridentate; lower margin of tooth 4 with small subsidiary cusps

### Key to the extant species of *Bathylasma*

1. Wall not flaring, alar growth line not parallel to inferior alar margin………..…………2

- Wall flaring, alar growth lines parallel inferior alar margin……………..*B*. *corolliform**e*

2. Basal width of CL1 < 33% that of CL2…………………………… …………………….3

- Basal width of CL1 33% to 50% that of CL2…………………………*B*. *chilense* sp. nov

3. Alar welting conspicuous, narrow …………………………………………….*B*. *alearum*

- Alar welting almost absent, very narrow…………….…………………………*B*. *hirsutum*

*Bathylasma chilense* sp. nov. not only represents the first record of the family in the Eastern Pacific, it is currently the deepest known species of the genus, albeit this record is surpassed by *Tetrachelasma southwardi* Newman & Ross, 1971 from the Antarctic Basin and off South America between 1190–2328 m, and by *T*. *tasmanicum* Buckeridge, 1999 from the South and East Tasman Rise between 2030–3600 m. The new species’ association with a zoantharian is also novel, as species of genus *Bathylasma* to date have only been cited as growing in rocks or on each other [3, 27], on the spines of echinoids of genus *Cidaris*, and in the demosponge *Topsentia novaezelandiae* [3]. The presence of sponges, anthozoans and several other species [34–36] in the hauls from where the new species was discovered indicates the presence of constant currents which, in addition to fallout from high surface productivity, may help this cold water fauna to thrive. High surface productivity due to upwelling is known to occur in the area [37].

A presumed *Bathylasma* sp. and two species of *Hexelasma* from deep waters in the subtropical Southwest Pacific were recently determined to nest in the Tetraclitoidea in the genetic study of Tsang et al. [17], confirming an alliance proposed by Newman & Ross [3] and Newman [16]. However, *Bathylasma* being under subtropical waters is unprecedented and therefore the specimens were sent us by B. K. K. Chan upon our request. As it turned out, not only were the specimens small for *Bathylasma*, but a calcareous basis could be seen on a few and therefore we returned them with the suggestion that they more likely represent specimens of *Mesolasma*, *Hexelasma*, or perhaps a new genus. It follows that the known range of *Bathylasma* in the southern hemisphere is apparently from Antarctica to as far north as 15°S under the divergence of the south-flowing East Australian from the Equatorial Current, and to 27°S under the Humboldt Current off Chile. The sole northern hemisphere representative of the genus, *Bathylasma hirsutum*, ranges in the NE Atlantic from the Faroes (62°N) south to the Azores and east to off the Straits of Gibraltar (35°N) [3, 11]. There are yet no records, fossil or extant, from the North Pacific.

A large proportion of the deep-sea thoracican barnacle species present in the Southeast Pacific are pedunculates (largely the Lepadomorpha and Scalpellomorpha), the only two symmetrical sessile barnacles (Balanomorpha) being *Solidobalanus nascanus* Zullo, 1964 and *Eochionelasmus paquensis* Yamaguchi & Newman, 1997 [38, 39]. This situation is similar in the adjacent territories of Antarctica, where of the 32 species found, the only balanomorph barnacle being the deepwater species *Bathylasma corolliforme* [3, 11].

Regarding the origin of the new species herein described, while an Austral West Wind Drift origin may seem most likely, we cannot ignore the Rapanuian or more western Indo-Pacific provinces via the seamounts and guyots of the Nazca and Sala y Gómez Ridge as stepping stones involved in their dispersion. The presence of several barnacle species of clear Indo-Pacific origins on an unnamed guyot in the Nazca Ridge [38] support this assertion. This can be tested by further sampling along the submarine features off northern Chile, coastal areas of which may include the easternmost representatives of some Indo-Pacific fauna [38–44]. On the other hand, in light of the extant North Atlantic population (*B*. *hirsutum*) and the U. Eocene record from North Carolina, the hypothesis that the bathylasmids are bipolar relicts of Tethys cannot be ruled out [43].
